# Influenza Illness and Partial Vaccination in the First Two Years of Life

**DOI:** 10.3390/vaccines9060676

**Published:** 2021-06-20

**Authors:** Abram L. Wagner, Lionel Gresh, Nery Sanchez, Guillermina Kuan, John Kubale, Roger Lopez, Sergio Ojeda, Eduardo Azziz-Baumgartner, Angel Balmaseda, Aubree Gordon

**Affiliations:** 1 Department of Epidemiology, School of Public Health, University of Michigan, 1415 Washington Heights, Ann Arbor, MI 48109, USA; awag@umich.edu (A.L.W.); jkubale@umich.edu (J.K.); 2 Sustainable Sciences Institute, Managua 14007, Nicaragua; lionel.gresh@gmail.com (L.G.); nerysanchez@icsnicaragua.org (N.S.); drakuan@yahoo.com.mx (G.K.); influenzacndr@minsa.gob.ni (R.L.); sojeda@icsnicaragua.org (S.O.); abalmaseda@minsa.gob.ni (A.B.); 3 Centro de Salud Sócrates Flores Vivas, Ministry of Health, Managua 12014, Nicaragua; 4 Laboratorio Nacional de Virología, Centro Nacional de Diagnóstico y Referencia, Ministry of Health, Managua 16064, Nicaragua; 5 Influenza Division, Centers for Disease Control and Prevention, Atlanta, GA 30333, USA; eha9@cdc.gov

**Keywords:** vaccine effectiveness, human influenza, fever, hospitalization, vaccination

## Abstract

More information about influenza in low- and middle-income countries could guide the establishment of pediatric influenza vaccine programs. This study (1) characterizes the burden of influenza in infants, and (2) compares signs and symptoms by prior influenza vaccination or influenza illness. Newborns from Managua, Nicaragua, were followed for two years. Data came from primary medical appointments, PCR testing, and parents’ daily symptom diaries. Logistic regression models estimated associations between preceding vaccination or illness and influenza incidence. Linear models compared duration of illness by prior vaccination or influenza illness. Among 833 infants, 31% had PCR-positive influenza, and 28% were vaccinated against influenza. Four (<0.5%) were fully vaccinated. Overall, influenza incidence was 21.0 (95% confidence interval (CI): 18.8, 23.2) per 100 person-years. Incidence was lower among those with prior influenza compared with those without preceding illness or vaccination (OR: 0.64, 95% CI: 0.44, 0.94). Partially vaccinated children had 1 day less fever than those without prior illness or vaccination (*p* = 0.049). A large proportion of children <2 years in Nicaragua contract influenza. Illness was attenuated for those partially vaccinated. Since few children were fully vaccinated, future studies will need to consider the effectiveness of a two-dose vaccination schedule.

## 1. Introduction

Infection with influenza virus can cause a variety of respiratory symptoms, and in its most severe presentation, influenza can result in acute lower respiratory infection, hospitalization, and death. Worldwide, there are between 291,243 and 645,832 deaths associated with seasonal influenza each year [1]. Children aged <5 years are at higher risk for complications and severe disease than older children and young adults [2]. Annually, there are an estimated 90 million new influenza cases [3] and between 9243 and 105,690 influenza-attributed deaths among children aged <5 years [1]. While the epidemiology of influenza has been well characterized in high income countries, 82% of influenza-attributed deaths among children occur in low- and middle-income countries (LMICs) [4]. Moreover, historically, most studies of influenza vaccine efficacy and effectiveness occur in high income countries [5].

Lack of information about the burden of influenza illness can limit the introduction of influenza vaccines in countries [6]. Within the United States, where the influenza burden and benefits of vaccination have been well characterized, the Advisory Committee on Immunization Practices currently recommends all individuals aged ≥6 months receive an annual influenza vaccination [7]. Likewise, the World Health Organization recommends certain high risk groups, including children aged 6 months to 5 years, receive influenza vaccination [2]. Nevertheless, influenza vaccines can be expensive, especially for LMICs and among children aged 6 months to 8 years who require two doses in their first year of vaccination [8]. More information about the epidemiology of influenza illness and the benefits of influenza vaccination would help guide investment in influenza vaccination in LMICs [9,10]. It is particularly important to understand the burden of influenza in a place like Nicaragua, where we previously found low perceived knowledge of influenza among mothers and relatively low intent to accept an influenza vaccine for their children [11].

Modeling studies suggest Nicaragua has a substantive burden of influenza [1]. Like many LMICs in the Americas, Nicaragua provides influenza vaccines free of charge to children 6–23 months of age to help mitigate this burden, but more information about influenza morbidity in Nicaraguan children would help policymakers understand the extent to which an influenza vaccination program is needed. It is also not known if the current program achieves full vaccination of the targeted children or if partially vaccinated children are conferred some protection against influenza illness. Using a well-defined cohort of infants in Managua, Nicaragua, who were followed for the first two years of life [12,13], this report aims to (1) better characterize the burden of influenza, and (2) compare influenza incidence in children aged <2 years by first documented influenza exposure (i.e., partial vaccination vs. prior influenza illness).

## 2. Materials and Methods

### 2.1. Study Population

Data were collected from the Nicaraguan Influenza Birth Cohort Study, a prospective cohort of infants enrolled at <4 weeks of age and followed until their second birthday, and who were enrolled from September 2011 through September 2014 in District II of Managua, Nicaragua [13]. Containing a population of 6.22 million, Nicaragua is a LMIC with a gross national income per capita of USD 2130 [14]. The study was based out of the Health Center Sócrates Flores Vivas (HCSFV), which in 2014 had a catchment area of approximately 61,411 people. This area contains both middle and lower class neighborhoods in terms of income and education levels, and includes some areas of urban slums [12].

### 2.2. Surveys and Sample Collection

Data from this study came from daily symptom diaries, annual health surveys, health care visits to HCSFV, and laboratory testing [12,13]. Parents filled out daily symptom diaries, indicating whether the child had rhinorrhea, cough, or fever. In health surveys conducted at enrollment, whenever the child moves household, or annually in March or April, families filled out a questionnaire about household socioeconomic status (SES). 

Study participants were offered primary health care and laboratory testing for free, and HCSFV was open every day for 24 h. Families were encouraged to bring their child to HCSFV at the first sign of subjective fever. Study participants who came to HCSFV had all medical information systematically collected and entered into study databases. Respiratory samples were collected from infants who met at least one of the following criteria: (1) fever (temperature ≥37.8 °C) or a history of fever and either rhinorrhea or cough; (2) fever or history of fever without a defined focus; (3) severe respiratory symptoms, including apnea, stridor, nasal flaring, wheezing, chest indrawing, or central cyanosis [15]; or (4) hospitalization for apnea, stridor, nasal flaring, wheezing, chest indrawing, central cyanosis, or sepsis. Respiratory samples were collected with two nasal and oropharyngeal swabs for children aged ≥6 months, and a single oropharyngeal swab for infants <6 months. Polyester-tipped plastic swabs were transported in tubes with 3 mL of viral transport medium and stored at a clinical laboratory in the HCSFV at 4 °C, and were transported to the National Virology Laboratory within 16 h (on weekdays) or 48 h (on weekends or holidays).

RNA was extracted from the swabs with a QIAamp Viral RNA Mini Kit (Qiagen Corporation, Valencia, CA, USA). Protocols from the United States Centers for Disease Control and Prevention (CDC) were followed to amplify, type, and subtype/genotype Influenza A and B viruses.

### 2.3. Outcomes

The primary outcome was laboratory-confirmed influenza, and, secondarily, the signs and symptoms associated with influenza. These signs and symptoms were abstracted from health center records. For the clinical symptom to be linked to influenza illness, the recorded date of either symptom initiation or fever initiation must have occurred 2 days prior to sample collection through to 30 days after (for pneumonia and acute otitis media) and from 2 days prior to sample collection through to 6 days after (for other symptoms: cough, rhinorrhea, vomiting, diarrhea, or rhonchi). Pneumonia was defined as cough and fast breathing (>60 breaths/minute for children aged <3 months, >50 breaths/min for children 3–11 months, and >40 breaths/min for children 12–23 months) [15]. We also summarized influenza severity into a dichotomous variable of mild vs. moderate-to-severe influenza [16], where moderate-to-severe influenza referred to influenza complicated by pneumonia, acute otitis media, or a fever >39 °C.

Calculations for the number of days of symptoms associated with an influenza illness were based on daily symptom diaries. To calculate the number of days of symptoms associated with an influenza virus infection, we summed the number of days with a symptom in the period of two days prior to the influenza infection through to 6 days after. 

### 2.4. Independent Variables

The primary independent variables were history of laboratory-confirmed influenza and influenza vaccination at least 14 days prior to the current influenza episode. All started with no history of influenza illness or vaccination but were recategorized as having a prior vaccination or a prior influenza illness if either of these events took place ≥14 days prior to another influenza event throughout the time series. For infants who were both vaccinated and had a history of illness, we categorized them based on which event (vaccination or illness) occurred first.

Nicaragua typically uses a Southern Hemisphere formulation of vaccines [17]. For all years where we have information on the vaccine used within the cohort (2013–2015), HCSFV stocked the trivalent Green Cross Southern formulation of the vaccine. However, it is possible that a limited number of children received another vaccine.

Other variables were added into the tertiles of household socioeconomic status (SES) constructed through an index which included whether the household had a car, a motorcycle, tap water, freezers or refrigerators, ≤4 persons per television, ≤2 persons per bedroom, ≥1 fan per room, a concrete, ceramic, or brick floor, along with not cooking with firewood. This method is comparable to how a wealth index is calculated in the Demographic and Health Surveys program [18]. Based on the annual surveys, we also extracted information about whether there was household crowding (≥4 persons per bedroom), or if the mother was currently breastfeeding the child. This measure of breastfeeding included both exclusive and non-exclusive breastfeeding.

### 2.5. Statistical Analysis

Descriptive statistics were generated from participant demographic and socioeconomic data. 

We included two sets of regression models in this study. The first was a longitudinal model measuring the incidence of influenza. We constructed multivariable logistic regression models with the outcome of laboratory-confirmed influenza infection with person-weeks as the denominator. We specified a mixed model with random effects per person. The main independent variable in this model was prior influenza illness or vaccination. Based on a priori considerations of confounders, we also included sex, age, household SES, smoker in household, household crowding, and breastfeeding status in the model. This model was limited to children ≥6 months (i.e., those age-eligible for vaccination).

Additionally, using multivariable models, we presented marginal estimates for a variety of clinical and parent-reported outcomes among influenza cases. These models only included laboratory-confirmed influenza cases. The outcomes were dichotomous measures of cough, rhinorrhea, vomiting or diarrhea, rhonchi, acute otitis media, moderate-to-severe influenza, and pneumonia, using logistic regression. Continuous outcomes included days of cough, fever, and rhinorrhea, using linear regression after testing for homoscedasticity and linearity. These models included only three independent variables: age, prior influenza illness or vaccination, and influenza type/subtype. The marginal estimates represent the average proportion of influenza cases with a given clinical characteristic or the average number of days with a certain symptom.

Significance was assessed at an α = 0.05 level. All analyses used SAS version 9.4 (SAS Institute, Cary, NC, USA).

## 3. Results

In total, 833 infants were enrolled into the study, with enrollment quotas for each month between 8 September 2011, and 5 September 2014 (Table 1). Participants could be followed until their second birthday and provided 73,498 person-weeks of data. Participants provided 88 weeks of data, on average, with 75% of the sample being followed at least through to 21 months of age. Overall, 216 (26%) had laboratory-confirmed influenza once, 38 (5%) twice, and 1 three times. The most common subtype was A/H3N2 (163, 55%), followed by A/H1N1 (50, 17%); 82 (28%) had a B strain. There was one A/H3N2-B/Yamagata co-infection. Most of the children (603, 72%) did not receive an influenza vaccine, and most of the rest (226, 27%) had only received one dose. Only four children (<0.5%) were considered fully vaccinated, i.e., having received two doses within one influenza season. None of the fully vaccinated children had influenza illness in the same season they were vaccinated. Almost all infants (806, 97%) were breastfed at some point in time. Many of the infants, 75% (622), were no longer being exclusively breastfed within two weeks of enrollment.

**Figure 1:**
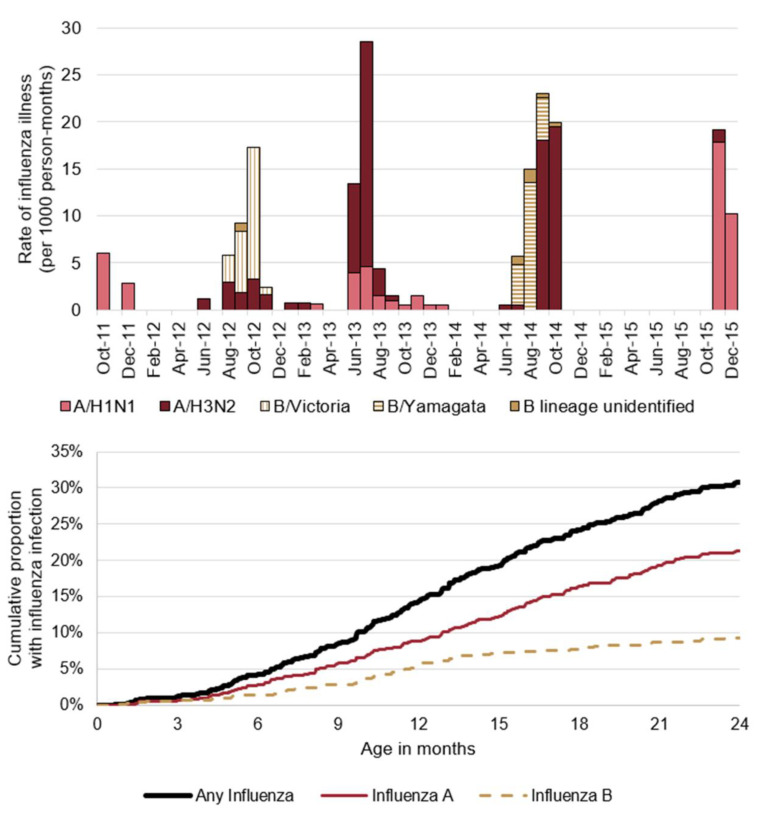
Monthly rates of influenza illness (upper panel), and cumulative proportion of influenza illness among infants <2 years of age in Managua, Nicaragua, 2011–2015 (lower panel). One A/H3N2-B/Yamagata co-infection during September 2014 is not portrayed.

During the first two years of life, a total of 296 influenza illness episodes were recorded in 256 children (31%) for a rate of 21.0 (95% CI: 18.8, 23.2) influenza episodes per 100 person-years (Figure 1 upper panel). By age, this rate was 6.0 (95% CI: 2.2, 9.9) episodes per 100 person-years for children <3 months, 12.9 (95% CI: 7.7, 18.1) per 100 person-years for children 3–5 months, 26.0 (95% CI: 20.9, 31.2) per 100 person-years for children 6–11 months, and 24.6 (95% CI: 21.1, 28.2) per 100 person-years for children 12–23 months.

By one year of age 14% of children had had an influenza virus infection (9% with influenza A and 6% with influenza B). By two years, this number increased to 31%: 21% with influenza A and 9% with influenza B (Figure 1 lower panel). Across the first two years, the rate of influenza-associated pneumonia was 1.1 (95% CI: 0.5, 1.6) episodes per 100 person-years, the rate of moderate-to-severe influenza was 2.2 (95% CI: 1.4, 3.0) episodes per 100 person-years, and the rate of influenza-associated hospitalization was 0.6 per 100 (95% CI: 0.2, 1.0) person-years.

We examined the association between influenza illness and individual and household exposures (Table 2). Compared with infants without influenza illness, those with history of a prior influenza illness had 0.64 times the odds of influenza (95% CI: 0.44, 0.94), but there was no difference between those with a prior (partial) vaccination. Current breastfeeding was also associated with reduced risk of influenza (OR: 0.65, 95% CI: 0.50, 0.83). We did not observe any significant differences by sex, age, household SES, or household crowding. 

Common clinical signs associated with the 295 influenza cases were cough (80%) and rhinorrhea (81%) (Table 3). A minority of children presented with more severe illnesses, like acute otitis media (6%), pneumonia (5%), or moderate-to-severe influenza (11%). On average, cases had 3.2 days of cough, 2.0 days of fever, and 3.7 days of rhinorrhea, according to parent-reported symptom diaries. Duration of fever varied by prior vaccination or illness: children without a prior influenza exposure had 2.0 days of fever, compared to 1.7 days among those with a prior natural infection and 1.1 days among those previously (partially) vaccinated (*p* = 0.049). 

Duration of cough and rhinorrhea varied by influenza type (Table 4). Cough lasted longer among influenza A cases (3.5 days for A/H1N1 and 3.4 days for A/H3N2, but only 2.3 days for all influenza B, *p* = 0.02), and there was a similar pattern for rhinorrhea (4.0 days for A/H3N2, 3.5 days for A/H1N1 and 2.9 days for influenza B, *p* = 0.04).

## 4. Discussion

Through a cohort study in a well-defined population, where primary care visits were provided for free to study participants, we found that almost one-third of all children had a symptomatic PCR-positive influenza virus infection before their second birthday. We found evidence that health outcomes after influenza illness varied by previous illness and vaccination status. Almost all vaccinated children were partially, and not fully, vaccinated, indicating difficulties in implementing a two-dose vaccination program. Nevertheless, we found evidence that even partial vaccination reduced duration of influenza illness. Additionally, although not the primary focus of our analysis, we found non-exclusive breastfeeding to be protective against influenza.

We found differences in influenza outcomes by preceding influenza illness and vaccination. Those with prior documented influenza illness had a lower risk of future influenza illness, but those with a vaccination had a shorter duration of fever, suggestive of vaccine-induced disease attenuation. Nevertheless, full vaccination (2 doses for children in their first season they receive the vaccine) results in better protection against influenza illness [19]. Moreover, vaccination of the mother, as well as other members of the household (“cocooning”), can help prevent disease in infants <6 months, who are too young for vaccination [20]. Due to changes in the predominant strain of influenza by year, the vaccine formulation will change [21]. Since the 2009 H1N1 pandemic and during the study period, the H1N1 pandemic strain co-circulated with H3N2 and B strains [22]. The low vaccine effectiveness in this study could be a function of a mismatch between the vaccine and circulating strains [23].

The overall rate of influenza in this population was similar or higher compared to other studies. The rate of influenza found in this study (21.0 per 100 person-years) was similar but slightly higher than the rate of influenza (15.5 per 100) from a preliminary analysis of this cohort study [13], possibly because of the types of influenza circulating in later years of the study. Comparatively, a study of influenza-associated illness in Peru found an incidence of 21.4 per 100 person-years among children <2 years [24], and 8 per 1000 among hospitalized children aged <6 months and 2/1000 among children aged 6–24 months in Buenos Aires, Argentina [25].

With the exception of previous studies conducted by our research group [13,26], there are few studies examining the range of clinical presentations after influenza virus infection in infants. One study from Wisconsin did contain a range of ages from children to adult [27], but many other studies focused on adults and particular populations (e.g., adults receiving transplants [28,29]), or limited their analysis to hospitalized patients. For example, studies in Kenya [30] and Rwanda [31] found that children <1 year had rates of influenza-associated SARI hospitalization of 0.1 and 0.3 per 100, respectively; rates notably lower than our rate of influenza-associated hospitalization (0.6 per 100 person-years). 

Local production of influenza vaccines in Central America and other LMICs could be valuable for several reasons. Within these areas, influenza is a substantial cause of hospitalization and deaths; with approximately 9505 hospitalizations (including 5595 children <5 years) and 382 deaths (including 64 in children <5 years) [32]. Local vaccine development could lower costs, and represent an investment in sustainable vaccination, particularly for influenza, which requires two doses for young children. Recently, a Russian pharmaceutical company has begun producing influenza vaccines in Nicaragua, and this could impact distribution of vaccines in the future. Having this vaccine product pre-qualified by the WHO would help procurement and regulatory review [33].

In our longitudinal analysis of influenza incidence, we controlled for breastfeeding and, in this exploratory analysis, found that breastfeeding protected against influenza illness (OR: 0.65, 95% CI: 0.50, 0.83). Previous studies found that breastfeeding increased production of type I interferons [34]. Schlaudecker et al. found that maternal influenza vaccination could increase influenza-specific IgA levels in breastmilk, with a corresponding decrease in respiratory illness among infants who were exclusively breastfed [35]. Our study’s findings offer support for a decrease in influenza illness, even among infants who were not exclusively breastfed. 

### Strengths and Limitations

This study has several strengths and limitations. Strengths include the longitudinal nature of the data and low loss to follow-up. We also have high confidence that we were able to catch the individual’s first illness with influenza by conducting active surveillance. Another strength of this study was the use of many different data sources, from clinical records, laboratory testing, and parental symptom diaries of symptoms; however, we acknowledge that parental symptom diaries may not be as reliable as clinical sources of information. Within the US, vaccine effectiveness varied widely in this period [36], from 19% in 2014–2015 [37] to 52% in 2013–2014 [38]. Another limitation of this study was that a low number of children were vaccinated. These low numbers limited our ability to determine type-, subtype-, or lineage-specific vaccine effectiveness. We also did not evaluate the impact of maternal immunization or infection history.

## 5. Conclusions

This longitudinal study of influenza in infants <2 years found a substantial burden of disease, with relatively high rates of infection and hospitalization. A minority of children were vaccinated, while very few were fully vaccinated. We found that partial vaccination still resulted in some protection against longer duration of illness, as measured by days with fever. We would expect additional protection against influenza illness if children had received a full two-dose schedule. Strengthened influenza vaccination programs for young children could be warranted in countries, like Nicaragua, which have a high burden of illness among young children. These programs should be capable of rolling out a two-dose schedule of vaccines, and this dispersal could be aided through local production of vaccines.

## Figures and Tables

**Table 1:** Characteristics of study participants in Managua, Nicaragua.

Characteristic	Category	Count	Proportion
Overall		833	100%
Year enrolled in study	2011	82	10%
	2012	255	31%
	2013	300	36%
	2014	196	24%
Sex	Female	418	50%
	Male	415	50%
Household socioeconomic status ^a^	Lower tertile	327	39%
	Middle tertile	245	29%
	Upper tertile	260	31%
Household crowding ^a^	≥4 people per bedroom	216	26%
Age of siblings ^a^	0–5 years old	541	65%
	6–12 years old	536	64%
	13–18 years old	447	54%
Age when breastfeeding stopped	<1 month	100	12%
	1–3 months	193	23%
	4–6 months	107	13%
	7–9 months	38	5%
	10–12 months	79	9%
	13–15 months	15	2%
	≥16 months	301	36%
Exclusively breastfed 6 months	Yes	163	20%
	No	670	80%
Vaccination completeness (for 1 season)	Never vaccinated	603	72%
	Partially vaccinated	226	27%
	Fully vaccinated	4	<0.5%
Vaccinated against influenza	No	603	72%
	1 dose	217	26%
	2 doses	38	5%
	3 doses	2	<0.5%
Symptomatic influenza infection	Never	578	69%
	1 time	216	26%
	2 times	38	5%
	3 times	1	<0.5%
Preceding influenza vaccination or influenza illness	No preceding vaccination or illness	429	52%
	Vaccination prior to any influenza illness	188	23%
	Influenza illness prior to any vaccination	216	26%

^a^ Value at baseline.

**Table 2:** Logistic regression model for influenza illness incidence among children 6–23 months old in Managua, Nicaragua (*N* = 53,750 person-weeks).

Characteristic	Category	OR (95% CI)	*p*-Value ^a^
Sex	Male	reference	0.8892
	Female	1.02 (0.80, 1.30)	
Age	6 to <12 months	1.02 (0.73, 1.42)	0.7156
	12 to <18 months	1.12 (0.82, 1.54)	
	18 to <24 months	reference	
Household socioeconomic status	Lower tertile	1.04 (0.77, 1.40)	0.7175
	Middle tertile	0.91 (0.67, 1.25)	
	Upper tertile	reference	
Household crowding	≥4 people per bedroom	1.01 (0.75, 1.36)	0.9335
	<4 people per bedroom	reference	
Child is currently breastfeeding	No	reference	0.0007
	Yes	0.65 (0.50, 0.83)	
Influenza vaccination or influenza illness in a preceding week	No preceding vaccination or illness	reference	0.0247
	Preceding vaccination	1.18 (0.83, 1.67)	
	Preceding influenza illness	0.64 (0.44, 0.94)	

Mixed model with random effects per person (full sample residual covariance estimate 1.0038, standard error 0.006123, limited sample residual covariance estimate 0.9988, standard error 0.01115). CI, confidence interval; OR, odds ratio, ^a^ Type III tests of fixed tests.

**Table 3:** Clinical and parent-reported outcomes (mean ± standard error) by preceding vaccination or illness for 295 ^a^ influenza episodes in a cohort of children <2 years in Managua, Nicaragua.

Characteristic	Total	Vaccinated	Influenza Illness	No Preceding Vaccination or Illness	*p* ^b^
Overall (count)	*n* = 295	*n* = 43	*n* = 55	*n* = 197	
Cough ^c^	80% ± 2%	87% ± 5%	81% ± 6%	84% ± 3%	0.7193
Rhinorrhea ^c^	81% ± 2%	89% ± 6%	66% ± 7%	80% ± 3%	0.0623
Vomiting or diarrhea ^c^	12% ± 2%	8% ± 5%	4% ± 3%	14% ± 3%	0.0901
Rhonchi ^c^	14% ± 2%	16% ± 6%	13% ± 5%	14% ± 3%	0.8893
Acute otitis media ^c^	6% ± 1%	3% ± 3%	1% ± 1%	8% ± 2%	0.1433
Moderate-to-severe influenza ^c^	11% ± 2%	12% ± 6%	8% ± 4%	12% ± 3%	0.6692
Pneumonia ^c^	5% ± 1%	8% ± 5%	5% ± 3%	3% ± 1%	0.4503
Days of cough ^d^	3.2 ± 0.2	3.1 ± 0.5	2.8 ± 0.4	3.4 ± 0.3	0.4758
Days of fever ^d^	2.0 ± 0.1	1.1 ± 0.3	1.7 ± 0.3	2.0 ± 0.2	0.0492
Days of rhinorrhea ^d^	3.7 ± 0.2	3.8 ± 0.5	2.9 ± 0.4	3.6 ± 0.3	0.2898

^a^ An A/H3N2-B/Yamagata co-infection is excluded. ^b^ Marginal estimates from mixed model with random effects per person. Logistic regression model for dichotomous outcomes, linear regression model for days. All models controlled for age, history of influenza illness and vaccination, and influenza type/subtype. ^c^ From clinical records. ^d^ From parent-reported symptom diaries.

**Table 4:** Clinical and parent-reported outcomes (mean ± standard error) by influenza type for 295 ^a^ influenza episodes in a cohort of children <2 years in Managua, Nicaragua.

Characteristic	Influenza A(H1N1)	Influenza A(H3N2)	Influenza B	*p* ^b^
Overall (count)	*n* = 50	*n* = 163	*n* = 82	
Cough ^c^	94% ± 4%	79% ± 4%	74% ± 6%	0.0589
Rhinorrhea ^c^	76% ± 7%	86% ± 3%	77% ± 6%	0.1554
Vomiting or diarrhea ^c^	8% ± 4%	9% ± 3%	7% ± 3%	0.8132
Rhonchi ^c^	23% ± 7%	14% ± 3%	9% ± 3%	0.1262
Acute otitis media ^c^	5% ± 3%	3% ± 2%	3% ± 2%	0.5928
Moderate-to-severe influenza ^c^	18% ± 6%	10% ± 3%	6% ± 3%	0.1705
Pneumonia ^c^	10% ± 5%	5% ± 2%	2% ± 2%	0.2265
Days of cough ^d^	3.5 ± 0.5	3.4 ± 0.3	2.3 ± 0.4	0.0240
Days of fever ^d^	1.5 ± 0.3	1.8 ± 0.2	1.4 ± 0.3	0.2588
Days of rhinorrhea ^d^	3.5 ± 0.5	4.0 ± 0.3	2.9 ± 0.4	0.0385

^a^ An A/H3N2-B/Yamagata co-infection is excluded. ^b^ Marginal estimates from mixed model with random effects per person. Logistic regression model for dichotomous outcomes, linear regression model for days. All models controlled for age, history of influenza illness and vaccination, and influenza type/subtype. ^c^ From clinical records. ^d^ From parent-reported symptom diaries.

## Data Availability

The data presented in this study are available on request from the corresponding author. The data are not publicly available due to personal information contained in the dataset.
